# Stereotactic radiosurgery with MLC‐defined arcs: Verification of dosimetry, spatial accuracy, and end‐to‐end tests

**DOI:** 10.1002/acm2.12583

**Published:** 2019-04-11

**Authors:** Ivan A. Brezovich, Xingen Wu, Richard A. Popple, Elizabeth Covington, Rex Cardan, Sui Shen, John Fiveash, Markus Bredel, Barton Guthrie

**Affiliations:** ^1^ Department of Radiation Oncology University of Alabama at Birmingham Birmingham AL USA; ^2^ Department of Neurosurgery University of Alabama at Birmingham Birmingham AL USA

**Keywords:** end‐to‐end verification, linear accelerator, stereotactic radiosurgery, TGN

## Abstract

**Purpose:**

To measure dosimetric and spatial accuracy of stereotactic radiosurgery (SRS) delivered to targets as small as the trigeminal nerve (TN) using a standard external beam treatment planning system (TPS) and multileaf collimator‐(MLC) equipped linear accelerator without cones or other special attachments or modifications.

**Methods:**

Dosimetric performance was assessed by comparing computed dose distributions to film measurements. Comparisons included the *γ*‐index, beam profiles, isodose lines, maximum dose, and spatial accuracy. Initially, single static 360° arcs of MLC‐shaped fields ranging from 1.6 × 5 to 30 × 30 mm^2^ were planned and delivered to an in‐house built block phantom having approximate dimensions of a human head. The phantom was equipped with markings that allowed accurate setup using planar kV images. Couch walkout during multiple‐arc treatments was investigated by tracking a ball pointer, initially positioned at cone beam computed tomography (CBCT) isocenter, as the couch was rotated. Tracks were mapped with no load and a 90 kg stack of plastic plates simulating patient treatment. The dosimetric effect of walkout was assessed computationally by comparing test plans that corrected for walkout to plans that neglected walkout. The plans involved nine 160° arcs of 2.4 × 5 mm^2^ fields applied at six different couch angles. For end‐to‐end tests that included CT simulation, target contouring, planning, and delivery, a cylindrical phantom mimicking a 3 mm lesion was constructed and irradiated with the nine‐arc regimen. The phantom, lacking markings as setup aids was positioned under CBCT guidance by registering its surface and internal structures with CTs from simulation. Radiochromic film passing through the target center was inserted parallel to the coronal and the sagittal plane for assessment of spatial and dosimetric accuracy.

**Results:**

In the single‐arc block phantom tests computed maximum doses of all field sizes agreed with measurements within 2.4 ± 2.0%. Profile widths at 50% maximum agreed within 0.2 mm. The largest targeting error was 0.33 mm. The *γ*‐index (3%, 1 mm) averaged over 10 experiments was >1 in only 1% of pixels for field sizes up to 10 × 10 mm^2^ and rose to 4.4% as field size increased to 20 × 20 mm^2^. Table walkout was not affected by load. Walkout shifted the target up to 0.6 mm from CBCT isocenter but, according to computations shifted the dose cloud of the nine‐arc plan by only 0.16 mm. Film measurements verified the small dosimetric effect of walkout, allowing walkout to be neglected during planning and treatment. In the end‐to‐end tests average and maximum targeting errors were 0.30 ± 0.10 and 0.43 mm, respectively. Gamma analysis of coronal and sagittal dose distributions based on a 3%/0.3 mm agreement remained <1 at all pixels. To date, more than 50 functional SRS treatments using MLC‐shaped static field arcs have been delivered.

**Conclusion:**

Stereotactic radiosurgery (SRS) can be planned and delivered on a standard linac without cones or other modifications with better than 0.5 mm spatial and 5% dosimetric accuracy.

## 1 INTRODUCTION

Stereotactic radiosurgery (SRS) is a valuable tool for the treatment of brain metastases, arterio‐venous malformations (AVM), and functional brain conditions like trigeminal neuralgia (TN) and vestibular schwannoma.^1^, ^2^, ^3^, ^4^ It is typically administered with dedicated equipment like the GammaKnife^®^ (GK) or CyberKnife^®^ that is available only at a few facilities.

Considering how many linear accelerators (linac) are available for conventional radiotherapy, extending their use to SRS could greatly expand access to that modality. Linac‐based SRS was introduced in the 1980s using standard treatment planning systems (TPS), occasionally supplemented by measured small‐field dose distributions. X‐ray jaw‐defined fields were used for delivery.^5^, ^6^, ^7^ Geometric and dosimetric accuracy was later augmented by in‐house developed software and accessories, as well as by fine‐tuning accelerators.^1^, ^2^, ^8^, ^9^, ^10^, ^11^, ^12^, ^13^

As technology evolved more precise linacs were made and special accessories and TPSs for SRS became commercially available.^4^, ^14^, ^15^, ^16^, ^17^ However, at institutions with limited resources the extra effort of commissioning and maintaining any additional system can be prohibitive. Substantial research has been devoted in recent years to circumvent the need for special apparatus by testing the suitability of multileaf collimators (MLC) and standard TPSs for SRS.

Dosimetric accuracy of small MLC‐shaped fields was investigated by numerous researchers. Poffenbarger et al^18^ studied the performance of the Eclipse and iPlan TPSs for square fields having side lengths as small as 2.5 mm, shaped by the HD120 leaf MLC. Film data agreed within 5% with the TPS for field sizes 7.5 mm or greater, but for smaller fields Eclipse underestimated the dose by 11% or more. Arcing fields showed similar behavior. Hrbacek et al^19^ compared dosimetric parameters from Eclipse with measurements for stationary beams of 1 × 1 cm^2^ and larger, delivered with a 120‐leaf MLC to a water phantom. Agreement between measurement and experiment was considered acceptable. Audet et al^20^ found the Eclipse Version 8.15.6 (Varian, Palo Alto, CA) TPS with Anisotropic Analytical Algorithm (AAA) algorithm sufficiently accurate for lesions greater than 7 mm in diameter and delivered the plans with a Novalis Tx accelerator (Varian) with 6 MV beams shaped by the HD120 MLC. Fog et al^21^ investigated accuracy of Eclipse versions 8.6 and 8.9 with grid spacings of 1.25 and 2.5 mm. Substantial inaccuracies for small fields were detected, especially if fields were only one to four leaves wide. The authors recommend caution with RapidArc plans because these may contain error‐prone small subfields, which cannot be controlled by the user. The newer Eclipse version and smaller grid spacing yielded better accuracy. Tanyi et al^22^ found dosimetric benefits of a 2.5 mm leaf MLC over a 5 mm system for SRS.

Spatial performance was addressed by Denton et al^23^ who investigated isocenter accuracy of a Novalis Tx and BrainLAB (Feldkirchen, Germany) system with ExacTrac and HD120 MLC. They recommend that daily congruence measurements between treatment isocenter (TIC) and other defined isocenters (couch, collimator, MLC, laser‐defined, gantry) should not exceed 1.25 mm. Huang et al^24^ studied targeting accuracy of four SRS systems using image guidance and found 0.2 mm agreement of cone beam computed tomography (CBCT) isocenter with TIC on the Edge accelerator (Varian Medical Systems, Palo Alto, CA). Sharpe et al^25^ investigated the stability of a CBCT system on an Elekta accelerator (Precise, Elekta Oncology Systems, Norcross, GA). Poffenbarger et al^18^ found spatial agreement between planned and measured arcing fields within 1 mm for the HD120 MLC and Eclipse TPS.

Collectively, the reports suggest that accurate small target SRS may be achievable with linacs and MLC‐defined fields. However, the papers address individual aspects of SRS and on a variety of accelerators and TPSs, leaving concern if all components would work coherently together on a single system. This paper presents a comprehensive series of tests of a widely used combination of linac and TPS before it was put into clinical service for treating very small targets.

We consider only arcs since cranial SRS is commonly administered with rotating fields. Potential error sources are minimized by using the more recent Eclipse Version 13.6.30 and the smallest available grid spacing of 1.0 mm, an MLC with 2.5 mm wide leaves, fields that are at least two leaves wide and prevent leaf motion by applying static arcs.

The investigation consists of three basic parts. Firstly we tested if the TPS could accurately predict dose distributions of small MLC‐shaped fields suitable for functional SRS (fSRS). Single arcs ranging from 1.6 × 5 to 30 × 30 mm^2^ were planned and delivered to a novel high‐precision block phantom containing radiochromic film in the coronal plane. The phantom had the approximate size of a human head and was equipped with markings that allowed better than 0.1 mm accurate setup on the accelerator by planar kV images. As a merit over commercial anthropomorphic phantoms that require setup based on CTs from simulation, our phantom isolated planning and delivery from potential upstream simulation errors.

In support of multiple non‐coplanar arc treatments the second part of the project investigated couch walkout under rotation, with and without a 90 kg load. A novel spherical pointer was constructed for mapping the trajectory of a point, originally positioned at CBCT isocenter, as the couch is rotated. The dosimetric effect of the measured walkout was then explored using the TPS and measurements.

The third part of the investigation involved end‐to‐end tests in compliance with ASTRO recommendations.^26^ These started with CT simulation of a cylindrical phantom having a 3 × 3 × 3 mm^3^ target replicating a TN treatment. The target was contoured and a treatment involving multiple arcs at different couch angles planned and delivered. The phantom contained film in the coronal and sagittal planes and had no marks or other setup aids. Similar to a patient, it was positioned for treatment using the six‐dimensional (6D) couch and automated matching of structural features. The level of agreement between planned and measured dose distribution was a gauge for expected clinical performance.

Consistency of the delivery method was examined by irradiating the block and the cylinder phantom 10 or more times during a 6‐month period. Performance of the block phantom and the cylindrical phantoms, in turn, was vetted by separate tests.

At the time of this writing static MLC‐defined 2.1 × 5 mm^2^ arcs at 5 equally spaced couch angles have been used to administer more than 50 rhizotomies of the TN and three thalamotomies under institutional review board (IRB) approved protocols.^27^, ^28^ In the TN treatments the target is the root entry zone, a maximum dose of 80 Gy is prescribed, and isocenter placed such that the 40 Gy surface abuts the brainstem. For thalamotomy the isocenter is placed at the ventral intermediate nucleus and 130 Gy prescribed as the maximum dose. Details about the fSRS treatments have been published elsewhere.^29^ The potential to generate specific non‐spherical dose distributions is demonstrated in this paper by a nine‐arc plan that closely resembles a GK with a 4 mm collimator.

In addition to its utility for fSRS with stationary arcs, our quality assurance (QA) method and block phantom have been used for more than 500 radiosurgeries delivered with VMAT and intensity modulated arc therapy (IMRT). These included primary brain tumors, metastases, and AVMs.

## 2 MATERIALS AND METHODS

Throughout this paper we use the IEC 61217 coordinate convention. For an observer standing at the foot of the treatment table and facing the gantry the positive x‐, y‐, and z‐ directions extend, respectively, left to right in the crossplane, toward the gantry along the caudal‐cephalad direction in the in‐plane, and toward the ceiling. The couch angle is 270° when the couch is in the 9 o'clock position, and increases with counterclockwise (CCW) rotation to 360°/0° as the home position is approached, and further increases from 0° to 90° when the couch reaches the 3 o'clock position.

All measurements were done on an Edge accelerator equipped with a 120‐leaf MLC (Varian Medical Systems, Palo Alto, CA). Dose distributions were computed on the Eclipse, Version 13.6.26 TPS with AAA algorithm provided by the same manufacturer.

### 2.A Description of the block phantom

**Figure 1:**
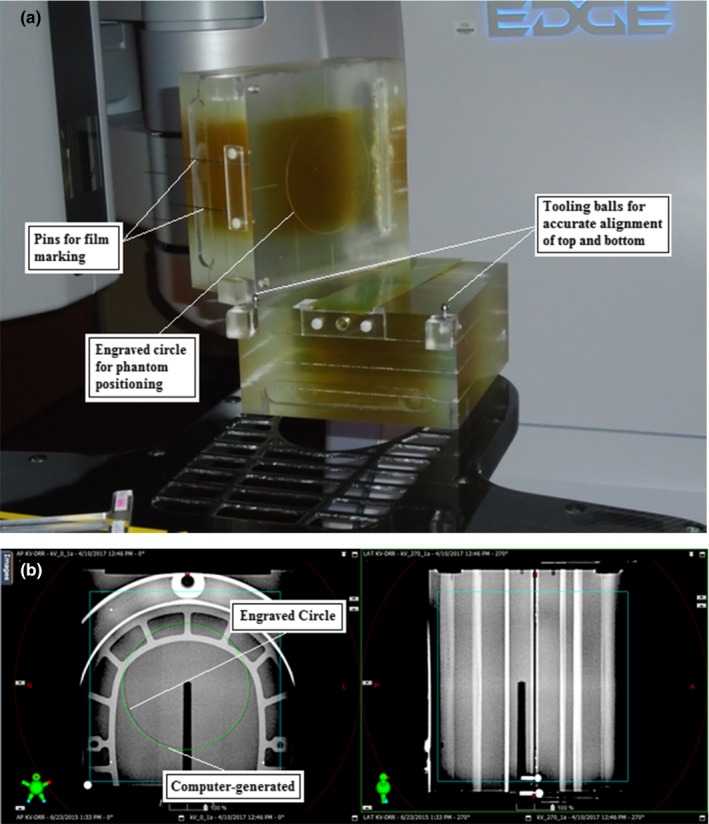
Block phantom. (a) Phantom with film in coronal plane. (b) Planar kV‐image guided setup of block phantom. Matching the engraved circle with a computer‐generated circular contour affords positioning at better than 0.1 mm.

The phantom was made of polymethylmetacrylate (PMMA), measured 18 × 19 × 17 cm^3^, and consisted of two approximately equal parts that allowed insertion of GafChromic EBT‐XD film (Ashland, Bridgewater, NJ) along the coronal plane [Fig. 1(a)]. For film marking the phantom was equipped with precisely milled channels that served as needle guides.

To allow accurate positioning on the accelerator, the upper section of the phantom had a 5 cm radius circular groove engraved at the film plane that was visible on anterior–posterior (AP) kV setup images. Matching the groove to a computer‐generated circle centered at kV isocenter afforded phantom positioning to better than 0.1 mm along the coronal plane [Fig. 1(b)]. In lateral kV images the junction between the upper and lower phantom sections was clearly visible as a sharp line. Matching this line to the digital cross hair allowed phantom setup along the vertical direction, also to better the 0.1 mm.

Films were digitized on an Epson Perfection V700 scanner and evaluated using an institution‐written script running on MatLab (The MathWorks Inc, Natick, MA). To account for potential variations in film sensitivity and accelerator output, calibration films were included with each set of measurements. The MatLab script displays maximum measured dose as well as overlays of computed and measured dose profiles and isodose lines. It also provides a *γ*‐analysis showing the percentage of pixels where *γ* > 1 under user‐selected criteria, typically 3% dose/1 mm distance to agreement. As a precise indicator of spatial accuracy, the analysis shows the distance by which the measured dose cloud has to be shifted along the x‐ and y‐directions for best agreement with the plan. The region of interest (ROI) for the analysis is the area within a 22.5 mm radius circle around isocenter where the dose exceeds 10% of the maximum dose. The match is considered optimal when the sum of the squared discrepancies between planned and measured dose for all pixels within the ROI is least. To reduce the effect of image noise, the maximum dose is defined as the average dose of pixels located within a 0.5 mm diameter circle encompassing the region of highest doses.

Calibration films covered the entire range of dose levels to be measured in the phantom. Doses were derived from a curve fit to the known transmission values of the calibration films recorded by the red color channel of the scanner. Calibration and measurement films were cut from the same sheet and oriented on the scanner along the same direction as recommended by the manufacturer of the radiochromic film.

### 2.B Verifying accuracy of block phantom

Spatial accuracy and repeatability of our experimental method were assessed by a series of radiation deliveries at well‐defined phantom positions. The phantom was placed at isocenter based on planar AP and lateral kV images. A planned dose of 8 Gy at isocenter was delivered with a 360° arc using a 2.4 × 5 mm^2^ field. The phantom was then moved in three equal steps, each step consisting of a −0.3 mm shift along the x‐direction and +0.4 mm shift along the y‐direction. At each position a film was taken and analyzed. The measured shift distances were compared to the known shifts. All shifts were applied from the console using the motorized drives and made in the same direction to avoid potential errors caused by backlash in the couch movement. Measured maximum doses at the starting position and the three shifted positions were compared to the plan.

### 2.C Assessment of TPS and accelerator accuracy for small rotating fields

To evaluate accuracy of the Eclipse TPS Version 13.6.26 with AAA algorithm for 10 MV flattening filter free (FFF) beams, we generated 360° arc plans using static MLC‐defined fields and compared these to film measurements using the block phantom. Field sizes of 1.6 × 5, 2.0 × 5, 2.4 × 5, and 5 × 5 mm^2^ were shaped by opening the central two leaves to the required size. Leaves in the central region of the high definition (HD) 120‐leaf MLC are, projected to isocenter, 2.5 mm wide. Larger fields up to 30 × 30 mm^2^ were generated by increasing leaf separations and opening additional leaves. The x‐ray jaws were set to 3 × 3 cm^2^ for MLC‐defined fields up to 20 × 20 mm^2^ and to 4 × 4 cm^2^ for the 25 × 25 and 30 × 30 mm^2^ fields. All irradiations were planned to deliver 8 Gy at isocenter, and were repeated 10 times at each field size.

The planning system was commissioned assuming a single point radiation source, 1.2% leaf transmission, and a dosimetric leaf gap of 0.86 mm, measured according to the sweeping gap method recommended by the manufacturer. Depth dose fractions and beam profiles were measured in a water tank covering the range of 3 × 3 to 40 × 40 cm^2^ fields. The “golden beam data” currently provided by the manufacturer were not available at the time of commissioning.

### 2.D Measurement of couch walkout

Even in a well aligned accelerator the couch axis may not pass exactly through isocenter and couch motion may not be perfectly smooth.^13^, ^23^ When the couch is rotated for multiple‐arc treatments the target shifts from its original setup position, resulting in potentially inaccurate dose delivery.

**Figure 2:**
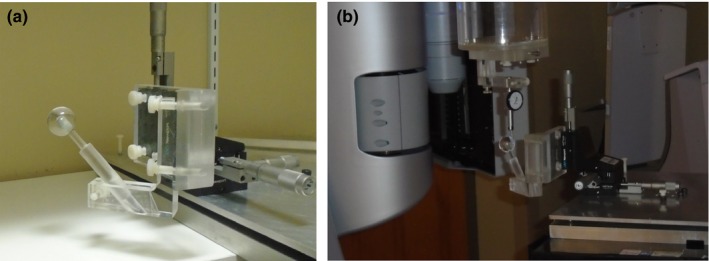
Ball pointer used for couch walkout measurements. (a) The pointer is positioned at cone beam computed tomography isocenter using linear translation stages. (b) A dial gauge mounted on the collimator and in contact with the surface of the pointer measures deviations of the ball as the couch is rotated.

To quantify walkout we built a novel pointer for mapping the trajectory of a target positioned at CBCT isocenter as the couch is rotated [Fig. 2(a)]. The pointer consisted of a 25.4 mm diameter. PMMA sphere with a concentric 6.35‐mm tungsten carbide sphere and was mounted on micrometer‐adjustable linear translation stages (Newport, Irvine, CA). The surface of the CBCT‐imaged plastic ball was not affected by artifacts cast by the metal ball that was provided for Winston‐Lutz (WL) tests.^9^ We were able to position the pointer with better than 0.1 mm accuracy at CBCT isocenter by matching its image to computer‐generated circles in the three principal planes, similar to the setup of our block phantom based on planar kV images. The <0.1 mm accuracy of pointer placement had been previously verified by applying known shifts to the pointer and comparison to offsets derived from CBCT images.^30^

A dial gauge attached to the collimator displayed deviations of the ball from CBCT isocenter at 0.01 mm resolution as the couch was rotated [Fig. 2(b)]. Measurements were done with no load and with a 90 kg stack of polystyrene plates placed on the couch to simulate treatment of a typical patient. The load was positioned such that its center of mass coincided with that of a patient undergoing SRS.

### 2.E Effect of couch walkout on dose distributions

The detrimental effect of walkout on the dose cloud of non‐coplanar arcs was investigated theoretically and experimentally. In the former method, walkout at angles used in a test plan was inserted into the TPS as isocenter shifts. Dose profiles of the so generated plan were compared to profiles of a plan that neglected walkout. The test plan was based on the block phantom. It consisted of nine non‐coplanar arcs of 2.4 × 5 mm^2^ MLC‐defined fields delivered at six different couch angles. Each arc covered 160°. Two arcs, 190° to 350° and 10° to 170°, were planned at table angles of 0°, 10°, and 350° while one arc was planned for 45°, 315°, and 90° couch rotation. Complete and 180° partial arcs were avoided to prevent hot spots at the anterior and posterior points of arc convergence.

Experimental testing involved planning the nine‐arc treatment for the block phantom without application of isocenter shifts, and delivery without couch corrections for walkout. Since this procedure assumed a perfect couch in planning whereas delivery involved walkout, agreement between plan and experiment was expected to be worse than in the single‐arc deliveries. However, based on the findings of the theoretical investigation, which suggested that couch walkout would have only a minor effect on the dose cloud, disagreement between plan and experiment should be only minor. The experiments were intended to verify that assumption.

### 2.F Description of the cylinder phantom for end‐to‐end tests

**Figure 3:**
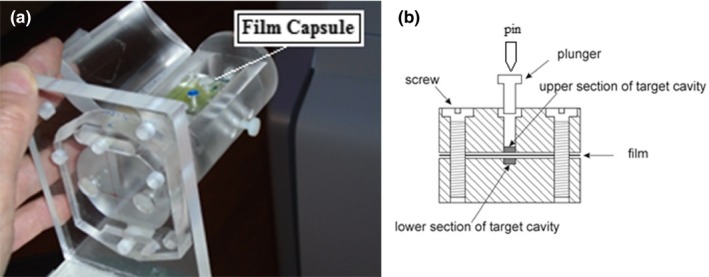
Cylinder phantom for end‐to‐end measurements. (a) Photograph of phantom. (b) Sketch of film capsule with air cavity as target and pin for film marking.

The phantom for the end‐to‐end test is shown in Fig. 3. It consisted of a 50 mm diameter cylinder of PMMA containing a groove for embedding a box‐shaped capsule. The capsule, measuring 24 × 24 × 38 mm^3^, was split along the mid‐plane for insertion of radiochromic film. It could be positioned within the cylinder having the film plane parallel to the coronal or the sagittal plane. A 1.5 mm deep 3.175 mm (1/8”) diameter cylindrical cavity was milled at the center of each section. When the two sections were joined the cavity simulated a 3 mm long 3.175 mm diameter lesion. Provisions were made for marking the film at the exact cavity center.

### 2.G Accuracy of measurements with the cylinder phantom

These tests were designed to assess the accuracy of three‐dimensional measurements made with the cylinder phantom. The phantom was small and light so that it could be mounted on precise linear translation stages that allowed phantom shifts at 0.01 mm accuracy. Initially the phantom was set up at isocenter by registering CBCT images to planning CTs. Accurately known shifts were then applied using the micrometer drives without other changes to the setup, thus avoiding experimental errors that could be introduced if shifts were done by repeated phantom setup. Radiation was delivered at each position twice, with film positioned along the coronal and the sagittal plane, respectively. Single 360° arcs of a 2.4 × 5 mm^2^ field were used.

Films were scanned and evaluated with ImageJ 1.48v, a program provided by the National Institute of Health.^31^ Phantom positions with respect to the TIC were derived from the distance between the pin prick marking cavity center and the centroid of the 50% isodose line that represented TIC. Distances between measured phantom positions were considered as measured phantom shifts, and were compared to the known applied shifts.

### 2.H End‐to‐end measurements with the cylinder phantom

The tests mimicked an intracranial treatment, starting at simulation followed by contouring, treatment planning, and delivery. Subsequent to simulation the target cavity was contoured and the nine‐arc regimen consisting of 2.4 × 5 mm^2^ arcs was planned to deliver 8 Gy at the cavity center. The electron density in the plan was set equal to PMMA density. Before irradiation, the air cavity was plugged with PMMA inserts to make it invisible on CBCT, mimicking a lesion identifiable on simulation CT but obscure to CBCT. The phantom was set up on the linac by matching CBCT images (head protocol, 100 kV, full rotation, 1 mm slices) to planning CTs of internal structures, similar to patient setup based on bony anatomy near the target. Automated registration and 6D couch movements were utilized, followed by a second CBCT to confirm accurate setup and, if necessary, small manual linear adjustments for optimal match. Radiation was delivered with marked films placed in the coronal plane and again with film in the sagittal plane. A third irradiation of unmarked film avoided dose errors in the vicinity of the pin prick.

Following scanning, films were evaluated with ImageJ. The distance between the centroid of the 50% isodose line and that of the prick mark at cavity center was the measure of spatial accuracy. All steps, including simulation, contouring, planning, and delivery were repeated ten times during a 6‐month period to evaluate stability. A second, independent method of film evaluation was used in later tests. It was based on a modification of the previously described MatLab script and provided overlays between plan and delivery in the coronal and sagittal planes.

## 3 RESULTS

### 3.A Verification of block phantom accuracy

Table 1 compares measured doses and phantom shifts to plans. The block phantom was initially set up at isocenter and relocated by three consecutive shifts, each −0.3 and 0.4 mm in the x‐ and y‐direction, respectively. At each of the four positions a single 360° 10 MV FFF arc was applied, planned to deliver 8 Gy at isocenter with a 2.4 × 5 mm^2^ MLC‐defined field. The measured doses and shift distances were derived from film located in the coronal plane.

**Table 1:** Block phantom performance. Doses in Gy, distances in mm. The shift errors *Δx* and *Δy* are the differences between the individual measured shifts distances from one position to the next minus the respective known distances

	Meas. dose	Planned position		Measured position		Shift error	
		*x*_*p*_	*y*_*p*_	*x*_*m*_	*y*_*m*_	*Δx*	*Δy*
Isocenter	8.05	0	0	−0.08	0.13	–	–
1 shift	8.02	−0.3	0.4	−0.37	0.58	0.01	−0.05
2 shifts	8.12	−0.6	0.8	−0.64	0.90	0.03	+0.08
3 shifts	8.07	−0.9	1.2	−0.88	1.37	0.06	−0.07
Average	8.07 ± 0.45%					0.03 ± 0.02	−0.01 ± 0.07

### 3.B Accuracy of the treatment planning and delivery system for single arcs

**Figure 4:**
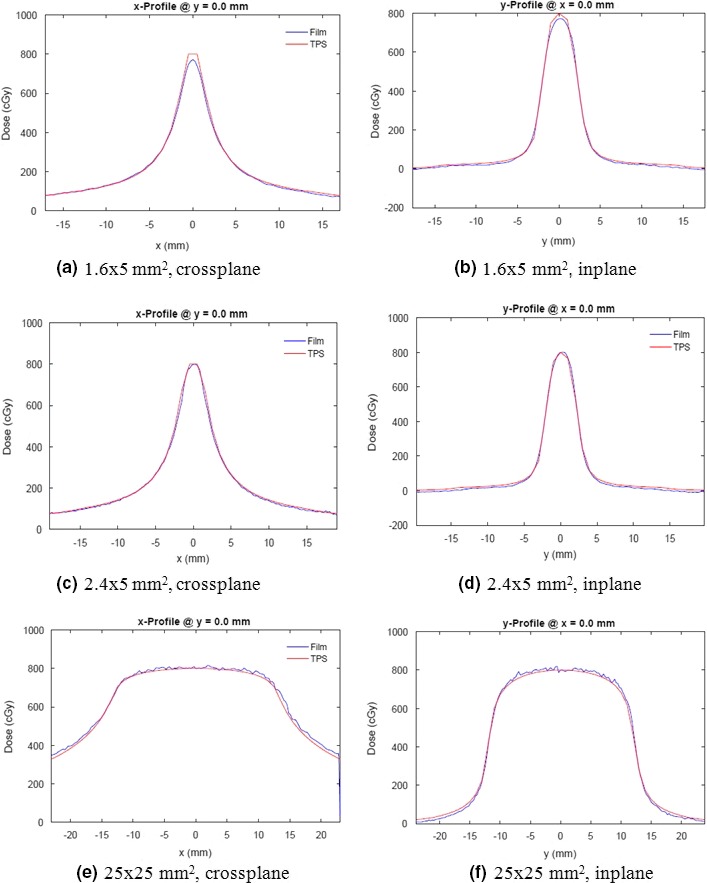
Comparison between calculated and measured dose profiles of stationary (multileaf collimator leaves do not move) 360° arcs. Delivery planned for 8 Gy to the block phantom with 10 MV flattening filter free beams. Measured isodose lines are shown in color, computed lines are in black. Data as measured, no shifts or dose scaling has been applied.

Figures 4(a)–4(f) compare planned and measured dose profiles produced by single arcs of three different field sizes. The profiles are shown as measured, they were neither spatially shifted nor was the dose normalized. The flat spots and sharp peaks of the computed dose profiles of the two narrower fields are due to the relatively coarse 1 mm computational grid, the smallest one available on the TPS.

A numerical comparison between plan and experiment is shown in Table 2 for a range of field sizes. Data in rows 1–9 represents averages of 10 measurements. The *γ‐*index is shown in the last column, while data more quantitative than the binary pass/fail *γ*‐analysis are presented in preceding columns.

**Table 2:** Comparison between planned and measured dose distributions in the block phantom. Single 360° arcs at various field sizes planned to deliver 8 Gy at isocenter using 10 MV flattening filter free beams (rows 1–9). Dose errors are positive (+) if the computed dose exceeds the measured dose. Targeting errors *Δx* and *Δy* are defined as the distances by which the center of the delivered dose cloud missed isocenter along the crossplane and the in‐plane, respectively. Positive values of *Δx* and *Δy* indicate, respectively, that radiation was delivered to the right (prone patient's left), and superiorly from kV isocenter. Two‐dimensional vector errors *v* defined as *v* = (*Δx* ^*2*^ + *Δy* ^2^)^0.5^. Row 10: Average of all 90 deliveries

Row	Field size (mm^2^)	MU	Average dose error (%)	Max dose error (%)	Average error *Δx* (mm)	Average error *Δy* (mm)	Average vector error *v* (mm)	Maximum vector error *v* (mm)	*γ*>1 3%/1 mm (%)
1	1.6 × 5	2701	3.7 ± 1.1	5.2	0.11 ± 0.09	0.07 ± 0.09	0.18 ± 0.05	0.23	0.00
2	2 × 5	2535	4.8 ± 2.6	9.0	0.09 ± 0.10	0.10 ± 0.11	0.17 ± 0.07	0.27	0.01
3	2.4 × 5	2388	2.8 ± 1.9	6.9	0.10 ± 0.11	0.08 ± 0.10	0.19 ± 0.08	0.28	0.00
4	5 × 5	1961	1.3 ± 1.2	2.8	0.09 ± 0.11	0.08 ± 0.11	0.19 ± 0.06	0.24	0.59
5	10 × 10	1452	0.5 ± 1.6	3.8	0.03 ± 0.13	0.06 ± 0.12	0.17 ± 0.08	0.27	1.05
6	15 × 15	1288	1.5 ± 1.4	4.4	0.05 ± 0.11	0.07 ± 0.11	0.16 ± 0.04	0.21	1.33
7	20 × 20	1212	1.8 ± 1.4	4.7	0.10 ± 0.12	0.10 ± 0.12	0.20 ± 0.08	0.29	2.30
8	25 × 25	1166	2.4 ± 1.1	3.8	0.01 ± 0.09	0.06 ± 0.09	0.14 ± 0.04	0.19	4.45
9	30 × 30	1138	2.9 ± 0.9	3.6	0.10 ± 0.12	0.08 ± 0.10	0.19 ± 0.08	0.33	6.41
10	All	n/a	2.4 ± 2.0	9.0	0.08 ± 0.11	0.08 ± 0.11	0.18 ± 0.07	0.33	n/a

### 3.C Couch walkout

**Figure 5:**
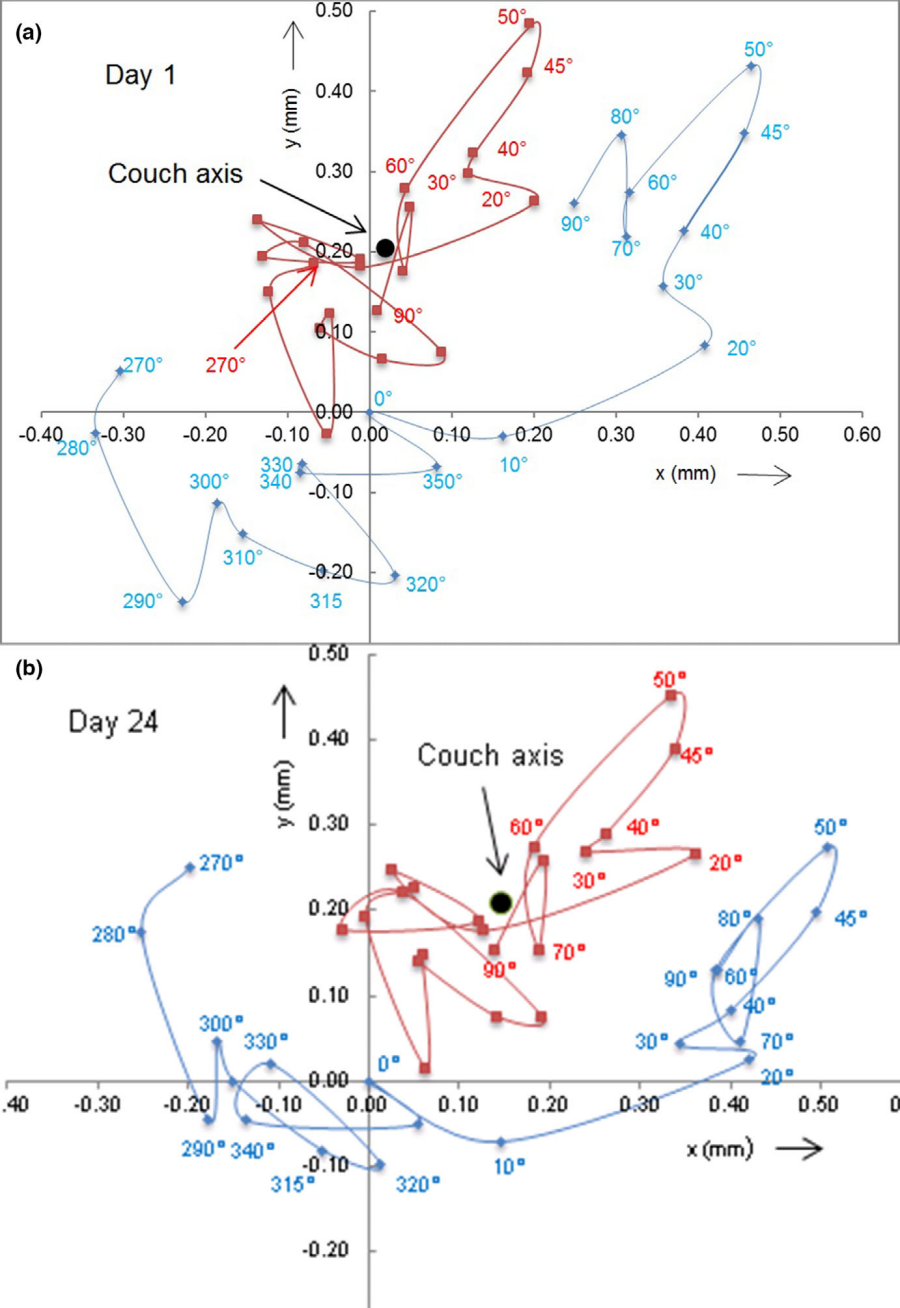
Couch walkout in horizontal plane as the couch is rotated. Blue rhombi depict the trajectory of the spherical pointer initially (couch angle 0°) positioned at cone beam computed tomography isocenter. Squares (red color) depict the (computed) wobble pattern that a pointer shifted to a position of least average walkout would trace. Measurement (a) and (b) taken 23 days apart.

Figure 5 shows two couch trajectories taken 23 days apart. The blue rhombi depict the trace of the spherical pointer positioned initially at CBCT isocenter at 0° couch angle as the couch was rotated counterclockwise to 90°, followed by a clockwise rotation to 270°.

Measurements were taken in 10° intervals and at 45° and 315°. The lines connecting the individual points indicate the sequence of pointer positions and may not represent the actual trajectories between points.

We interpret the convoluted pointer trajectories as consisting of two components, a circular path around the couch axis and superimposed wobble. The axis is defined as a vertical line passing through a point in space from which the average distance (averaged over all measured couch angles) to a pointer placed at the “optimal” position on the couch is minimum. The position of the couch axis and that of the optimally placed pointer were found by an iterative method. After mapping the trajectory of a pointer originally placed on the couch at CBCT isocenter, the average distance of all pointer positions from the center of mass of the trajectory was found. As a first iteration, it was assumed that the pointer was shifted on the couch by a given vector *v*_*1*_. The trajectory of the shifted pointer was then calculated by first transforming *v*_*1*_, which rotated with the couch, to the room coordinate system. Application of the transformed vector to each pointer position of the original track yielded a new trajectory. As before, we calculated the average distance of the individual pointer positions from the center of mass of the new track and compared it to that of the original track. The next iteration repeated the procedure, but with assumption of a slightly different shift vector *v*_*2*_. The procedure was repeated until a shift vector *v*_*optimal*_ was found, which minimized the average distance between pointer positions and the center of mass of the track. The center of mass of the optimized track defined the couch axis. The vector extending from the original pointer position at CBCT isocenter to the couch axis is a measure of misalignment between CBCT isocenter and couch axis. The track of a pointer placed on the couch at the optimized position is considered as wobble due to an imperfect couch suspension (Fig. 5, red squares).

The similarity between the patterns in Figs. 5(a) and 5(b) caused by couch wobble (red squares) suggests that couch motion, albeit not perfectly smooth, changes little with time. We attribute the differences between the measured tracks (blue rhombi) to day‐to‐day variations in the position of CBCT isocenter with respect to the couch axis.

We experimentally verified the validity of the analysis by recording the trajectory of a pointer, originally positioned at CBCT isocenter as the couch was rotated. From the trajectory we computed *v*_*optimal*_ and the shape of the wobble pattern that would result if the pointer was placed at the optimal positon. We applied *v*_*optimal*_ to the pointer using the micrometer stages and repeated the trajectory measurement. All points of the so‐found track agreed within better than 0.1 mm with the computed wobble pattern. Vertical excursions of the pointer, indicative of vertical couch walkout, were less than ±0.05 mm and considered negligible.

**Figure 6:**
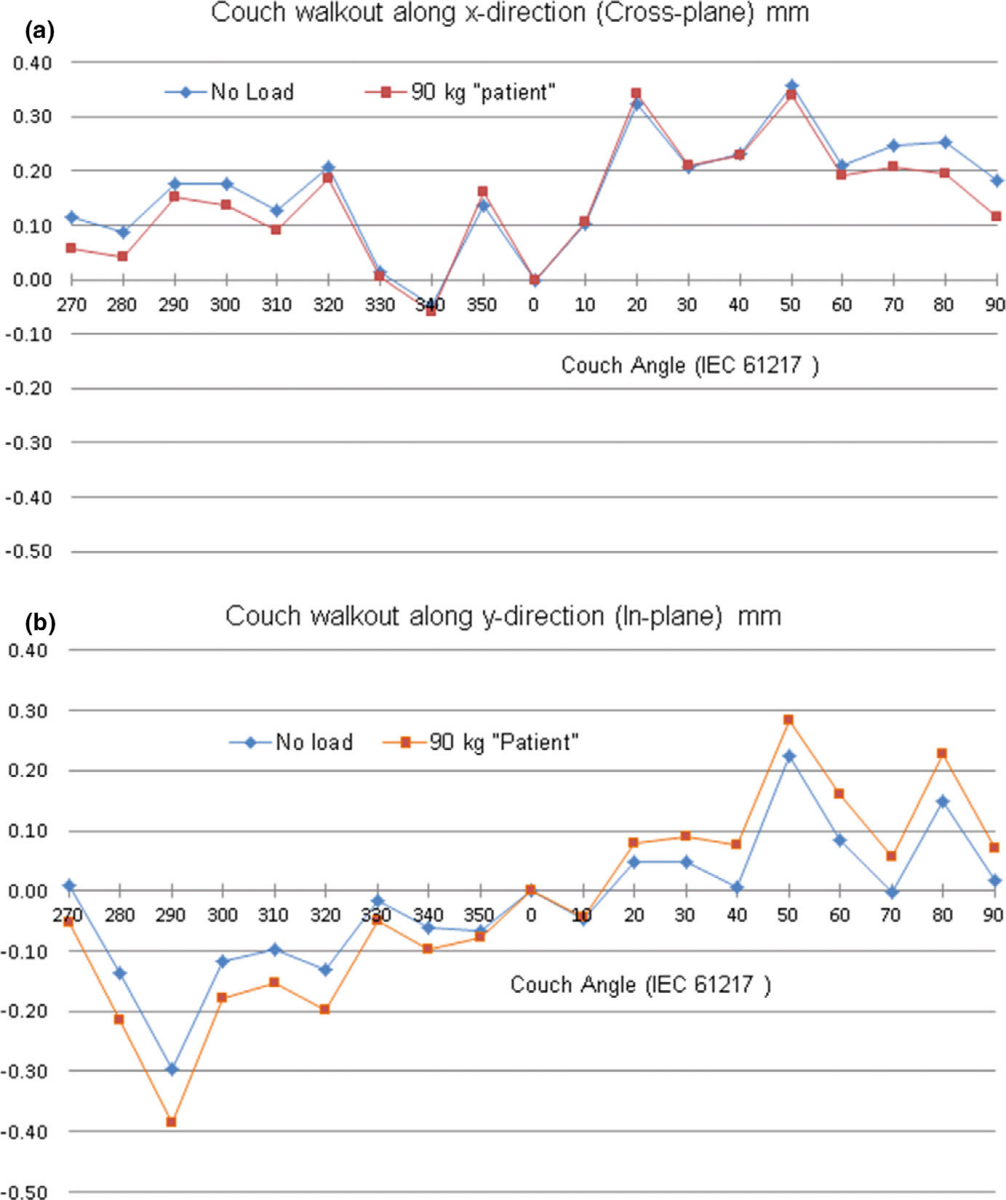
Walkout of a pointer with no extra load and with a 90 kg load placed on the couch. Pointer positioned at cone beam computed tomography at the beginning of each test. (a) Walkout along the crossplane. (b) Walkout along the in‐plane.

The effect of load on walkout is shown in Fig. 6. In the first measurement the track of a pointer, originally positioned at CBCT isocenter, was mapped with no extra load on the couch (blue rhombi). When a 90 kg load was placed on top of the treatment table, the couch top sagged, causing the pointer to shift by −0.3, 0.9, and −4.9 mm along the x‐, y‐, and z‐directions, respectively. After repositioning the pointer to CBCT isocenter the track was re‐mapped (red squares). Since the difference between the tracks was within the limits of reproducibility, the effect of load was considered minimal and all consecutive measurements were made with no extra load on the couch. There was no measurable change of walkout along the vertical direction.

### 3.D Effect of couch walkout on dose distributions

**Figure 7:**
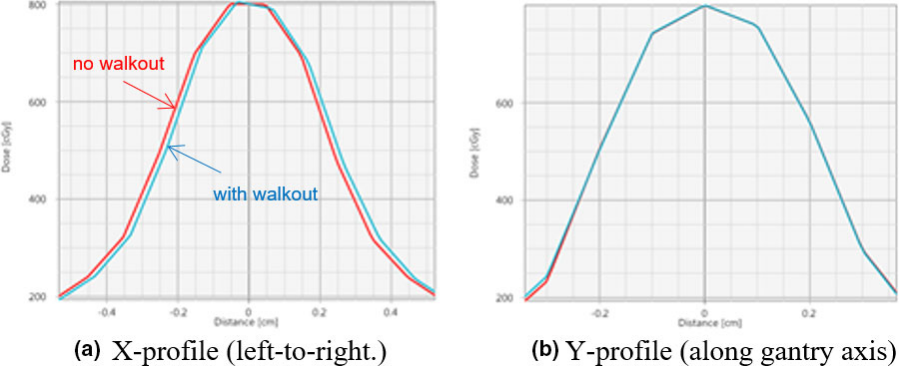
Comparison between x‐ and y‐dose profiles computed with no couch walkout and with walkout taken into account. (a) Couch excursions move the dose cloud by about 0.16 mm from isocenter toward the right and make it slightly asymmetric. (b) Couch excursions have negligible effect on the y‐profile.

The couch walkout shown in Fig. 5(a) was applied in the nine‐arc treatment plan for the block phantom as isocenter shifts of the respective arcs. We chose this trajectory because it was the largest one encountered during a 1‐month monitoring period and therefore would show the largest dose errors. Figure 7 shows the effect of the couch deviations by comparing computed dose profiles that incorporate the shifts to profiles of a perfect couch (no wobble, couch axis agrees perfectly with CBCT isocenter).

**Figure 8:**
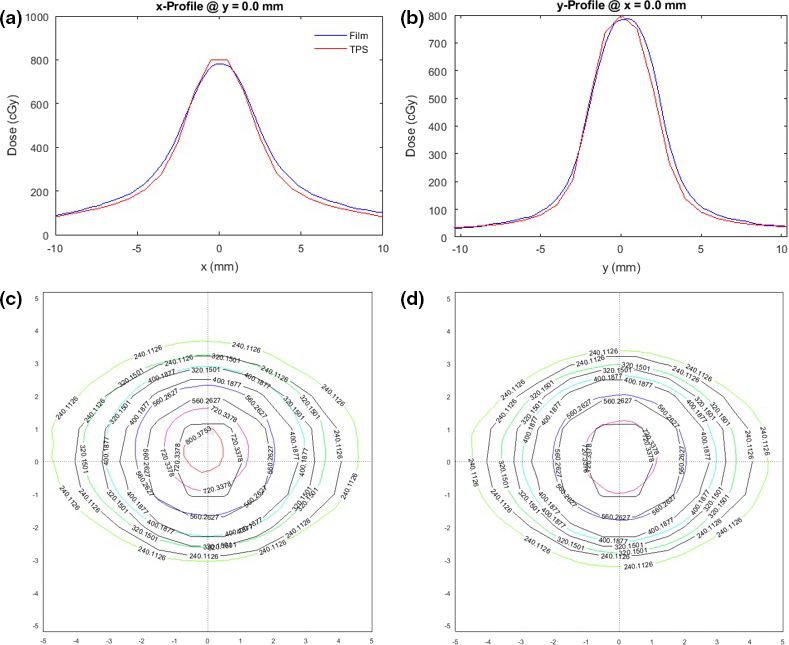
Comparison between calculated and measured doses in the coronal plane. 8 Gy was planned for delivery with 10 MV flattening filter free beams to the block phantom, using nine non‐coplanar 2.4 × 5 mm^2^ arcs. Couch walkout was neglected during planning and delivery. (a), (b) Dose profiles, and (c) isodose lines as measured. (d) Measured isodose lines scaled by a factor of 1.021 and shifted by −0.03 and −0.14 mm in the x‐ and y‐directions, respectively, for best match. Measured isodose lines (30, 40, 50, 70, 90, and 100%) in color, computed lines in black.

According to the theoretical investigation couch walkout should have only a minor effect on the dose cloud. Based on that finding it would not be necessary to correct for walkout on our accelerator. We verified this hypothesis by planning a treatment with the nine‐arc configuration without consideration of couch walkout and delivery with the couch that exhibited the measured walkout. A comparison between plan and measurement is shown in Figure 8 and Table 3. Only the maximum vector error of 0.43 mm (Table 3, next to last column) is slightly larger than the 0.28 mm error of single arcs (Table 2, row 3), confirming that the detrimental effect of couch walkout is only minor.

**Table 3:** Quantitative comparison between planned and measured dose distributions of non‐coplanar arcs delivered to the block phantom. Nine 160° arcs at six different couch angles were planned to deliver 8 Gy at isocenter using 10 MV flattening filter free beams. Data are averages of 10 measurements, defined as in the caption of Table 2

Field size (mm^2^)	MU	Average dose error (%)	Max dose error (%)	Average error *Δx* (mm)	Average error *Δy* (mm)	Average vector error *v* (mm)	Maximum vector error *v* (mm)	*γ*>1 3%/1 mm (%)
2.4 × 5, 9 arcs	2477	0.3 ± 2.0	3.1	0.05 ± 0.13	0.13 ± 0.10	0.20 ± 0.08	0.43	0.14

### 3.E Verification of cylinder phantom accuracy

Table 4 compares measured phantom shifts to known shifts applied with micrometer‐driven translation stages. Note the close agreement between applied and measured shift distances. It not only signifies an accurate phantom and experimental procedure but also a highly repeatable dose delivery by the accelerator. Any change in beam geometry between consecutive deliveries would appear as a discrepancy between applied and measured shifts.

**Table 4:** Performance of cylinder phantom. Planned dose of 8 Gy delivered with single arcs of 2.4 × 5 mm^2^ fields, firstly with phantom positioned at isocenter, then at four different positions shifted by known distances from the previous positions. Applied shift distances along the three cardinal coordinate directions are shown in columns 2–4. Corresponding film measurements are shown in columns 5–7. Shift errors, defined as differences between measured and applied shifts are shown in the last three columns

Shift #	Applied shift (mm)			Measured shift (mm)			Shift error (mm)		
	*x*_*a*_	*y*_*a*_	*z*_*a*_	*x*_*m*_	*y*_*m*_	*z*_*m*_	*Δx*	*Δy*	*Δz*
1	0.20	0.20	−0.20	0.26	0.24	−0.15	0.06	0.04	0.05
2	−0.40	−0.40	0.40	−0.36	−0.44	0.38	0.04	−0.04	−0.02
3	0.20	0.20	−0.20	0.21	0.17	−0.26	0.01	−0.03	−0.06
4	0.30	0.00	0.00	0.27	0.00	0.04	−0.03	0.00	0.04
						Average	0.02	−0.01	0.00
						Stdev.	0.03	0.03	0.04
						Avg. error, all shifts		0.005	
						Stdev., all shifts		0.04	

### 3.F End‐to‐end measurements with cylinder phantom

**Figure 9:**
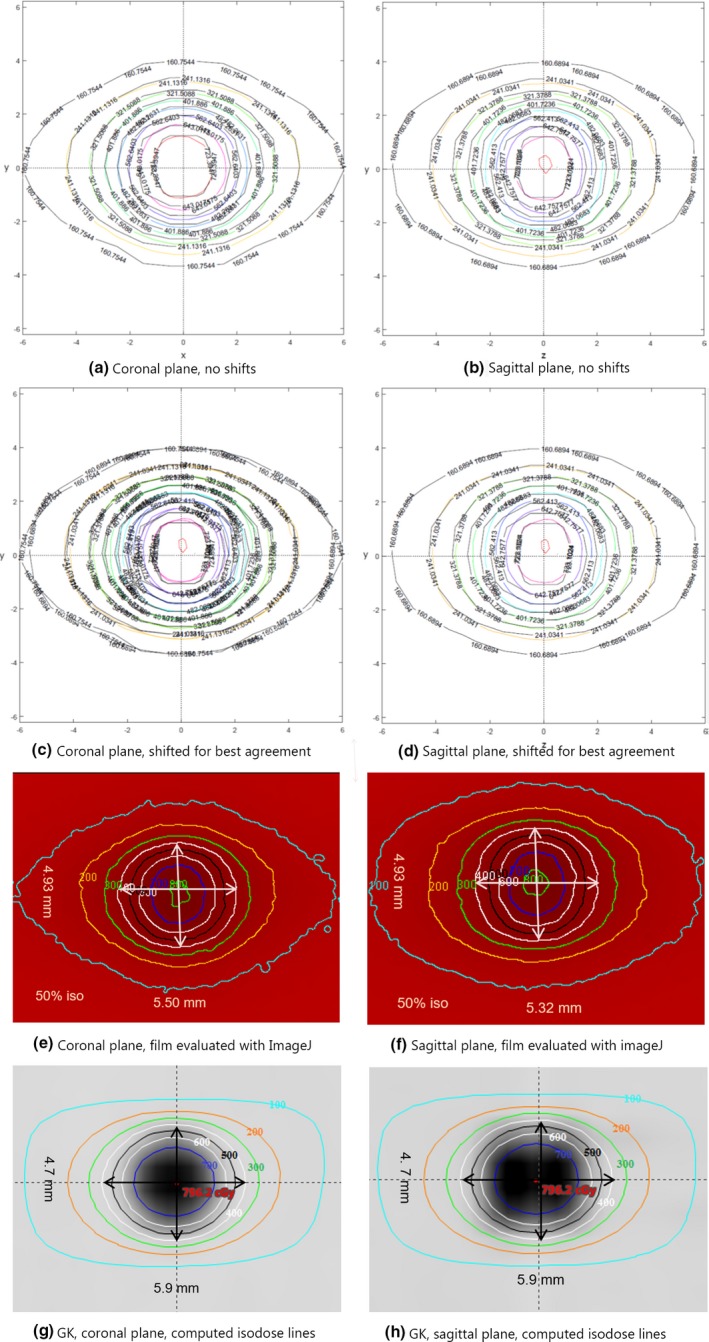
Computed and measured isodose lines in the coronal (left column) and the sagittal plane (right column) of the cylinder phantom. Doses of nine‐arc 2.4°5 mm^2^ plan evaluated using MatLab (a–d) and ImageJ (e and f). Computed doses of a GammaKnife^®^ (GK) with 4 mm collimator are shown for comparison (g and h). For the nine‐arc regimen the widths of the 50% isodose lines in the x‐, y‐, and z‐directions are, respectively, 5.50, 4.93, and 5.32 mm. The corresponding widths for the GK are 5.9, 4.7, and 5.9 mm.

Figure 9 shows an overlay of measured and computed isodose lines in the coronal (left column) and sagittal (right column) planes due to the nine‐arc plan. The overlay was generated by an in‐house script running on MatLab. Top row (a) and (b) depicts raw measurements, while the measured doses shown in the second row have been shifted and normalized for optimal fit. Isodose lines are shown in 10% increments from 20% to 90%. Measured lines are in color, planned lines in black. The measured dose cloud in the coral plane (c) was shifted by 0.04 mm along the x‐direction and 0.12 mm along the y‐direction. No dose normalization was applied. In the sagittal plane (d) the shifts were 0.15 mm and −0.02 mm along the y‐ and z‐direction, respectively. The measured dose was multiplied by a normalization factor of 0.997 for best fit. In both planes the gamma index of the original uncorrected data, defined as 3% dose/0.3 mm distance to agreement, was <1 within the ROI that included all pixels with doses >15% of maximum. Isodose lines from films evaluated with ImageJ are shown in figures (e) and (f). Computed dose distributions of a GK with 4 mm collimator are included for qualitative comparison, (g) and (h).

A numerical evaluation of maximum dose‐ and targeting errors measured with the cylindrical phantom is shown in Table 5. The films were analyzed using ImageJ. Ten separate CT simulations were done, each followed by contouring the target cavity, planning for 8 Gy at isocenter, and delivery. Each test involved three separate irradiations, one with marked film in the coronal plane and one with marked film in the sagittal plane for positional measurement, followed by irradiation of unmarked film in the coronal plane for dose assessment.

**Table 5:** Comparison between planned and delivered doses. Dose errors positive if planned dose exceeds measured dose. Positive values of *Δx* and *Δy* and *Δz* indicate, respectively, that radiation was delivered to the right (prone patient's left), superiorly, and anteriorly from CBCT isocenter. Three‐dimensional vector error *v* defined as *v* = (*Δx* ^*2*^ + *Δy* ^2^ + *Δz* ^*2*^)^0.5^

MU	Average dose error (%)	Max dose error (%)	Average error *Δx* (mm)	Average error *Δy* (mm)	Average error *Δz* (mm)	Average vector error *v* (mm)	Maximum vector error *v* (mm)
1592	1.2 ± 2.3	3.8	−0.18 ± 0.14	0.04 ± 0.18	−0.03 ± 0.10	0.3 ± 0.10	0.42

## 4 DISCUSSION

Our phantom measurements have shown that radiation treatments can be planned and delivered with MLC‐shaped rotational fields as small as 1.6 × 5.0 mm^2^ with dosimetric and spatial accuracy well within the limits recommended by pertinent professional organizations.^26^, ^32^, ^33^, ^34^ Two phantoms of different sizes and two different methods of film analysis were used in the tests. Accuracy of our phantoms and method of measurement, in turn, was also verified.

In the clinic, the MLC‐shaped fields were found practical in more than 50 fSRS treatments. We use 2.1 × 5 mm^2^ arcs at five equally spaced couch angles to obtain near‐spherical dose distributions.^29^ A dose cloud resembling a GK with 4 mm collimator is presented in this paper to show how customized dose distributions can be planned and delivered with MLC‐defined static arcs.

We attribute the higher accuracy of our dose computations primarily to better modeling of the MLC leaves by the newer Eclipse version 13.56 TPS with AAA algorithm vs the earlier versions 8.5 through 8.9 investigated by other researchers.^20^, ^21^ A whitepaper by Varian^35^ supports our assumption. According to the paper accurate results can be obtained with 6 MV beams for static MLC‐defined 5 × 5 mm^2^ fields and 20 × 20 mm^2^ jaw openings using AAA versions 10.0 and 11.0. It suggests leaving the jaws at the 3 × 3 cm^2^ position and to use the MLC to shape the smallest apertures, the approach we took in our research. Our work shows that version 13.56 yields accurate computations for even smaller fields and 10 MV FFF beams. Furthermore, we avoided potential error sources pointed out in the literature. The stationary fields used in our work prevent leaf motion and its associated inaccuracies that afflict IMRT and VMAT treatments. We use a small grid spacing of 1.0 mm in dose computations, deliver radiation with the high‐definition MLC, and shape fields by opening two or more leaves.

Our in‐house built QA tools proved valuable in daily routine. In addition to its use in this research and fSRS, the block phantom has been employed in more than 500 patient‐specific QA tests for IMRT and VMAT deliveries to targets that were too small for verification with our diode‐based commercial phantom. Couch walkout is readily checked with the spherical isocenter pointer.

The vector error of only 0.18 mm in the single‐arc deliveries to the block phantom is noteworthy as it is smaller than the longitudinal central ray (CR) shifts under gantry rotation revealed by WL tests. Shifts of ±0.4, ±0.21, and ±0.25 mm were observed, respectively, by Gibbs et al on a Varian Clinac 2100 C,^11^ by Denton et al on a Varian/Novalis accelerator,^23^ and in our own work on the Edge. We believe that the rotational application of radiation averages deviations at individual gantry angles, causing a small widening of beam profiles but having little effect on the center of the radiation cloud. Our measured dose profiles matched the planned ones accurately, demonstrating that the effect is only minor.

The slightly larger targeting errors in the nine‐arc delivery compared to single‐arc delivery were likely caused by couch walkout. Dose errors due to walkout could be eliminated by shifting the couch or the MLC as the couch is rotated.^1^, ^15^, ^36^ However, on our accelerator the resulting dose error was minimal, allowing us to neglect walkout during planning as well as delivery. A 90 kg load had no measureable effect on walkout, in agreement with the paper by Schmidhalter et al.^37^

End‐to‐end tests with the cylinder phantom revealed positional errors of 0.30 ± 0.10 mm in average, 0.42 mm maximum. Inaccuracies during simulation, contouring, and setup may have contributed to the slightly larger targeting error compared to the block phantom. For comparison, corresponding errors of the C‐model GK were 0.367 ± 0.126 and 0.626 mm, respectively.^38^

Although the in‐house phantoms were not anthropomorphic, we feel that they were well suited for the given task. The block phantom had the approximate dimensions of a human head and was valuable in establishing dosimetric accuracy of the TPS and spatial accuracy of the delivery system. Pixel averaging in the images provided by the large circular outline and matching with a computer‐generated circle allowed reproducible phantom setup at kV isocenter to better than 0.1 mm. (We were unable to more precisely quantify setup accuracy because the digital display on the linac console provides only 0.1 mm resolution). Setup based on planning CTs, typical for commercial phantoms, may be less reproducible. Potential flaws introduced during simulation are carried along as systematic errors and may be incorrectly interpreted as flaws in the delivery system.

Except for the absence of intra treatment motion, our end‐to‐end tests with the cylinder phantom were representative of patient treatment. Tests included all steps encountered in a patient treatment, beginning with CT simulation followed by target contouring, planning, setup on the accelerator, and delivery. The phantom had no scribe marks or other setup aids and required positioning by matching the surface and internal structures of CBCTs to planning CTs. The 3 mm cavity replicated the small targets typical in fSRS, a feature not available in commercial anthropomorphic phantoms. The target cavity was plugged during delivery, simulating treatment of a lesion that is not visible on CBCT.

Our initial concern about continued accuracy was largely dispelled by the QA tools provided by the manufacturer. Congruence of kV imaging isocenter and 6 MV TIC is assured by the automated IsoCal alignment procedure.^39^, ^40^ IsoCal uses a phantom containing 16 BBs and takes more than 100 views with kV and MV beams at various gantry and collimator angles. After establishing TIC, computer‐driven mechanical shifts are applied to the kV imaging panel as the gantry is rotated, correcting for flex of gantry and imaging system arms. IsoCal also provides a QA check, “Isocenter Verification” that displays the maximum misalignment between imaging panels and the projection of the TIC encountered at any gantry angle. Following calibration by IsoCal, misalignment is typically <0.1 mm and can worsen to about 0.2 mm in the course of a few days. If misalignment exceeds our departmental limit of 0.2 mm IsoCal is run to restore accurate alignment.

Accuracy and stability of the MLC positions, a most critical requirement for small‐field delivery was demonstrated by Stevens et al.^41^ Analyzing log files of 178 treatment fields the investigators found typical and maximum leaf positioning errors of 0.01 and 0.04 mm, respectively. They concluded that an appropriately commissioned TPS can be used for accurate and clinically appropriate design of trigeminal neuralgia treatment plans utilizing a HD‐MLC.

Congruence of the 10 MV FFF isocenter with imaging isocenter—we use 10 MV FFF for all SRS cases—is attained during accelerator installation by matching all photon beams to the 6 MV beam. The recently introduced machine performance check (MPC)^42^, ^43^, ^44^ allows continued monitoring of congruence. MPC uses the same phantom as IsoCal and displays maximum couch walkout, position of jaws and individual MLC leaves, in addition to dosimetric data such as output constancy and beam flatness. Individual x‐ray jaw offsets in relation to the jaw‐defined beam axis are provided for 6 MV. For the 10 MV FFF beam only the “collimator shift,” defined as the shift of the jaw‐defined beam axis relative to a previously established baseline, is shown. Nevertheless, a variation of collimator shift from the baseline would alert the physicist about changes of the 10 MV FFF beam that may require machine realignment.

Our QA method also provides for expedient tests following repairs or adjustment of critical components. For example, we re‐mapped couch walkout after the linac was repositioned for closer match between couch axis and CBCT isocenter. According to our test, the adjustment moved the CBCT isocenter along the positive y‐direction from its original position of 0.35 mm inferior of the couch axis to 0.15 mm superior. The engineers who had done the adjustment confirmed a 0.5 mm shift of the accelerator along the +y‐direction, in agreement with our measurement. We also found that the realignment reduced maximum couch walkout by 0.15 mm.

Because of the heightened demands on accuracy in fSRS, we run IsoCal and MPC the day before treatment and perform WL tests at a multitude of gantry and couch angles. These are in addition to the daily WL spot checks done at a few selected gantry angles. We also measure the output of a single arc using a scintillator. While arc treatment with stationary MLCs would normally not require patient‐specific QA checks, nevertheless we carry out such tests with the block phantom as another safeguard. Finally we want to point out that, while our TPS provides accurate output based on the original commissioning data, other TPSs may require adjustment of input data for optimal small‐field calculations.

## 5 CONCLUSIONS

Small‐field irradiation suitable for SRS can be reliably planned and delivered with stationary MLC‐defined arcing fields using a modern TPS and linac without special software or hardware. Because of the critical nature of SRS an especially vigorous QA program is recommended. With the increasing number of modern accelerators in the field SRS should become more readily available.

## CONFLICT OF INTEREST

Dr. Popple reports grants and personal fees from Varian Medical Systems, outside the submitted work; In addition, Dr. Popple has a patent Systems and Methods for Providing Radiotherapy Treatment US Patent Application No. 62/025,165 with royalties paid by Varian Medical Systems. None of the other authors report potential conflict of interest.
